# The use of mechanistic evidence in drug approval

**DOI:** 10.1111/jep.12960

**Published:** 2018-06-11

**Authors:** Jeffrey K. Aronson, Adam La Caze, Michael P. Kelly, Veli‐Pekka Parkkinen, Jon Williamson

**Affiliations:** ^1^ Nuffield Department of Primary Care Health Sciences University of Oxford Oxford UK; ^2^ School of Pharmacy The University of Queensland Brisbane Australia; ^3^ Department of Public Health and Primary Care, School of Clinical Medicine University of Cambridge Cambridge UK; ^4^ Department of Philosophy and Centre for Reasoning University of Kent Canterbury UK

**Keywords:** causality, epistemology, evaluation, evidence‐based medicine, philosophy of medicine

## Abstract

The role of mechanistic evidence tends to be under‐appreciated in current evidence‐based medicine (EBM), which focusses on clinical studies, tending to restrict attention to randomized controlled studies (RCTs) when they are available. The EBM+ programme seeks to redress this imbalance, by suggesting methods for evaluating mechanistic studies alongside clinical studies. Drug approval is a problematic case for the view that mechanistic evidence should be taken into account, because RCTs are almost always available. Nevertheless, we argue that mechanistic evidence is central to all the key tasks in the drug approval process: in drug discovery and development; assessing pharmaceutical quality; devising dosage regimens; assessing efficacy, harms, external validity, and cost‐effectiveness; evaluating adherence; and extending product licences. We recommend that, when preparing for meetings in which any aspect of drug approval is to be discussed, mechanistic evidence should be systematically analysed and presented to the committee members alongside analyses of clinical studies.

## 1 INTRODUCTION

This paper has two aims. First, to highlight the fact that mechanistic evidence *
Note that the term “mechanistic evidence” is ambiguous. It can mean evidence of the existence or details of the operation of mechanisms, or it can mean taking previously established mechanisms as evidence to adduce other conclusions.^1^ In this paper, we distinguish between these two possibilities by using explicit terminology: “evidence *for* mechanisms” to refer to the former and “evidence *from* mechanisms” to refer to the latter. We use the term “mechanistic evidence” when we refer to both types of evidence, *for* and *from* mechanisms. informs the drug approval process in a wide variety of ways—a point that tends to be under‐appreciated and under‐described in the evidence‐based medicine (EBM) literature. Secondly, to argue that drug approval processes should explicitly include mechanistic evidence as part of the assessment of manufacturers' applications for licences and in postmarketing surveillance, so that it can be appropriately scrutinized and, if need be, challenged.

This paper contributes to the general programme of making the role of mechanistic evidence in the health sciences more transparent and methodologically rigorous, so that decisions can be grounded in evidence. EBM has sought to improve health outcomes by making evaluation of clinical studies more rigorous. However, the results of clinical studies form only part of the evidence base. In particular, good quality evidence of mechanisms can be obtained from a wide variety of sources, not just clinical studies (Table 1). The EBM+ programme seeks to clarify the role of such evidence and make its evaluation more explicit, in the hope that considering these important forms of evidence in conjunction with the results of clinical studies can lead to further improvements in health outcomes.^2^, ^3^, ^4^, ^5^

**Table 1:** Sources of evidence for mechanisms

Direct manipulation: eg, in vitro or ex vivo experiments
Direct observation: eg, biomedical imaging, autopsy
Clinical studies: eg, RCTs, observational studies, case reports
Confirmed theory: eg, biochemistry
Analogy: eg, animal experiments
Simulation: eg, agent‐based models

Drug approval is a “hard case” for the thesis that one should explicitly scrutinize evidence for mechanisms. This is because it is common EBM practice to hold that when randomized studies are available, as they almost always are in the case of drug approval, they should be considered in preference to—or even to the exclusion of—other kinds of evidence. As we shall argue, this is not always appropriate: randomized studies may not measure (or may be underpowered to measure) outcomes of interest, either beneficial or, especially, harmful, and in any case other evidence, eg, from mechanisms, can support or undermine the results of such studies. Either way, mechanistic evidence should always be taken into consideration.

This paper is structured as follows. First, in Section 2, we outline the ways in which evidence for mechanisms explicitly informs the drug approval process, through the phased approach to approval, including animal studies and human clinical studies. The roles of such evidence in these processes are well recognized. In other tasks related to drug approval, the roles of evidence for mechanisms are also crucial, but less well recognized and often implicit. In the rest of the paper, we show that evidence for mechanisms is relevant to all the tasks that are important in drug approval: evaluating the efficacy of a drug; evaluating harms; evaluating the external validity of a claim about a study population; determining drug usage; extending the licence of a drug; evaluating the quality of a formulation; evaluating adherence; and evaluating cost effectiveness. Finally, in Section 12, we draw some conclusions.

Before proceeding, it will be useful to define the key concepts to which we appeal, to avoid ambiguity.

A *complex‐systems mechanism* is a complex arrangement of entities and activities, organized in such a way as to be regularly or predictably responsible for the phenomenon to be explained.^6^ An example of a complex‐systems mechanism is the heart's mechanism for pumping blood. A *mechanistic process* consists of a spatio‐temporal pathway along which certain features are propagated from the starting point to the end point.^7^ An example of a mechanistic process is the process by which a signal is propagated from an artificial pacemaker to the heart. We use the term *mechanism* to refer to either a complex‐systems mechanism, or a mechanistic process, or some combination of the two. For example, the mechanism for pumping blood might be constituted by the complex‐systems mechanism of an artificial pacemaker for producing a timing signal, the complex‐systems mechanism of the heart itself, and the mechanistic process linking the two.

A *clinical study* for the claim that *A* is a cause of *B* repeatedly measures the values of a set of measured variables that includes *A* and *B*. In an *experimental study*, the measurements are made after an experimental intervention. If no intervention is performed, the study is an *observational study*.

A *mechanistic study* for the claim that *A* is a cause of *B* is a study that provides evidence of the details of the mechanism by which *A* is hypothesised to cause *B*. Note that a clinical study for the claim that *A* is a cause of *C*, where *C* is an intermediate variable on the mechanism from *A* to *B*, is also a mechanistic study for the claim that *A* is a cause of *B*, because it provides evidence of the details of the mechanism from *A* to *B*. A clinical study for the claim that *A* is a cause of *B* is not normally a mechanistic study for that claim*,* because, although it can provide indirect evidence that there exists some mechanism linking *A* and *B*, it does not normally provide evidence of the structure or features of that mechanism.

We emphasize here, as footnoted earlier, that *evidence for mechanisms* includes evidence of either the existence of a mechanism or evidence of the details of a mechanism. While mechanistic studies provide evidence of the details of a mechanism, clinical studies can provide evidence of the existence of a mechanism. Thus, high quality evidence *for* mechanisms can be obtained by a wide variety of means, as shown in Table 1.^3^

A claim of *effectiveness* is a claim that a particular causal relationship holds in some target population of interest. A claim of *efficacy* is a claim that a particular causal relationship holds in some specific study population under particular controlled conditions. A claim of *external validity* (or *applicability*) is a claim that a particular causal relationship holds more widely than in a specific study population, controlled clinical setting, or experiment. Effectiveness is often established by establishing efficacy in a study population and then establishing external validity to a target population of patients.

## 2 CLINICAL DRUG DISCOVERY AND DEVELOPMENT

In the drug discovery process, mechanistic evidence is widely acknowledged to be crucial. Contemporary drug discovery and development are exceedingly “target driven” (see section 7, Harms), starting with the characterization of a biological component that can serve as an intervention point to a disease mechanism, and proceeding to the design and synthesis or biological production of a compound able to interact with the target component. Once manufactured, a new compound needs to be evaluated for beneficial and adverse effects. This process is typically divided into phases (Table 2).

**Table 2:** An outline of typical phases of clinical drug development

Phase 0	Phase 1	Phase 2	Phase 3	Phase 4
Preclinical studies/microdose pharmacology	Single‐dose and multiple‐dose “safety” studies	Studies over the target dose range in patients	Studies of efficacy and adverse events	Postmarketing studies

In the pre‐clinical phase (phase zero), a compound is tested in animals to determine an appropriate dose for human trials and to characterize any major organ toxicity. In phase I, the compound is tested in healthy human volunteers, unless the drug is likely to have adverse effects that obviate this (eg, drugs used to treat cancers). In phase II, the compound is tested in a small number of patients affected with the targeted disease. Phase I to midway through phase II is focused on learning what doses of the drug are tolerated and how the drug affects major organ systems, and confirming that the drug is likely to be effective for the proposed indication, called “proof of concept” (typically: a randomized trial showing that drug *A* improves some interim measure *C,* a so‐called biomarker, which is an indicator that the drug is likely to benefit the clinically relevant measure *B*). In phase III, the compound is tested in larger human trials in patients who have the disease. The latter part of phase II until the completion of phase III is focused on learning how best to use the drug in patients (determining appropriate dosing and learning how different patient characteristics influence dosing) and confirming that the drug is efficacious and sufficiently free of common harms in a sample of target patients.

Phase III trials, so‐called pivotal trials, are conducted for regulatory approval and seek to confirm that the drug benefits patients on a clinically relevant outcome measure, which may be a biomarker or a direct measure of improvement of the disease. The design of these trials is informed by what has been learned throughout the drug's development. For instance, phase III trials will test the doses of the drug that have been identified in late phase II trials, and the selection of participants will be informed by what has been learned about the benefit to harm balance so far assessed (eg, patients with renal or hepatic impairment or those taking other medicines that may interact with the experimental treatment may be excluded from the trial).

A successful phase III trial is a basis for applying for a marketing authorization (the official term for the licence). Sometimes, approval is conditional on conducting further studies after approval, typically to monitor unexpected adverse effects or reactions.

Sheiner characterized clinical drug development as a series of “learn‐confirm” cycles.^8^ *Learning* components of clinical drug development seek to answer key questions about the drug and its actions on the body. Examples of these questions include: What are the mechanisms by which the drug enters the body, distributes throughout the body, and is cleared from the body? What doses of the drug are pharmacologically active? With what biological systems does the drug interact and how does it affect these systems? The answers to these questions are determined by establishing the complex‐systems mechanisms and mechanistic processes at play. This knowledge then informs the design and interpretation of clinical studies, which seek to *confirm* that the drug is efficacious and sufficiently safe in the study population.

What is understood about the actions of the drug gets progressively more sophisticated, and the evidence regarding its clinical benefits more compelling, as it successfully progresses through clinical development. This is due to the interplay between the emergence of evidence for mechanisms and confirming that the drug benefits patients in rigorously designed randomized trials. While Sheiner focused on clinical drug development, it is important to note that this interplay does not stop at drug approval. The questions that consumers, clinicians, and regulators have about medicines go beyond the evidence provided by even the most compelling phase III randomized trials. Will the drug benefit a *specific* consumer given his or her characteristics? Is the drug effective in the kinds of patients who present to the clinic? Is the drug safe in patients with impaired renal or hepatic function, and what dose is appropriate? Are particular age groups at greater risk of adverse reactions? While partial answers to these questions will be provided by the studies conducted during the drug's development, it is important that both mechanistic evidence and evidence of clinical outcomes continue to evolve, especially given that clinical use of the drug will extend beyond the groups of patients represented in the original studies.

There is an increasing focus on appropriate evaluation throughout a drug's life‐cycle. To do this well, to appropriately answer the questions of consumers, clinicians, and regulators, it is necessary to have reliable and relevant evidence for mechanisms and clinical outcomes. This goal is undermined by a tendency to define the best evidence for drug evaluation solely in terms of what provides the best evidence of clinical outcomes. In later sections, we provide specific examples of how mechanistic evidence informs the evaluation of drug efficacy and safety. Further examples are provided to demonstrate how drug evaluation benefits from making such evidence more explicit. Key benefits of this include better informing the interpretation and generalization of randomized trial results and identifying mechanistic assumptions that require further scrutiny.

## 3 PHARMACEUTICAL QUALITY

Pharmaceutical tests are conducted to demonstrate that the drug, and its specific formulations, are sufficiently stable for clinical use. Key aspects that need to be determined are the rate at which the drug product loses potency and the identification and properties of any degradation products. The shelf life/expiry date of a drug product is determined by considering the rate at which potency is lost and the presence and toxicity of any degradation products. In the absence of toxic degradation products, the expiry date of a drug product is typically set such that the drug will retain greater than 90% of its labelled potency for the duration of its shelf life under recommended storage conditions.

Knowledge and evidence of mechanisms play a central role in ensuring and assessing the stability of drug formulations. Knowledge of the chemical characteristics of the drug inform the way that the drug will be formulated and stored. Key stability tests are undertaken on the medicinal product in the selected storage container when stored as recommended. For example, glyceryl trinitrate (nitroglycerin) is highly volatile, and tablets tend to lose potency over time. Glyceryl trinitrate tablets need to be stored in glass containers with a foil lined cap, because loss of potency will be exacerbated if the tablets come in contact with plastic or other permeable packaging material; cotton wool, often included in drug containers, must not be packaged with glyceryl trinitrate tablets.

The general approach to stability testing has developed in response to developments in the understanding of mechanisms of degradation. Stability tests assess degradation of the medicinal product over time under variations in temperature, humidity, and light. Two key stability tests include a long‐term test under recommended storage conditions (eg, 12 months with the product stored at either 25°C or 30°C and a relative humidity of 65%) and an accelerated test (eg, 6 months at 40°C and a relative humidity of 75%). The long‐term test provides information on degradation under recommended storage conditions and the accelerated test provides information on degradation when the product is stored outside recommended conditions. It is important to understand likely degradation under deviations from recommended conditions, because some deviation is likely during the shelf life of the product—during storage by the manufacturers/wholesalers, distribution to pharmacies and hospitals, and the far less well controlled storage conditions provided by consumers.^9^

Finally, specific stability tests conducted for a drug product depend on the particular physiochemical properties of the drug and what is known or learned about the specific mechanisms of degradation to which the drug is susceptible. Stress tests early in drug development are important in providing evidence of degradation pathways and providing an opportunity to test degradation products for clinical and adverse effects. This information guides decisions regarding appropriate storage and appropriate stability testing. This is especially important for biologic medicines, because of the concern that degradation products may cause an immune response. Another example of mechanisms of degradation informing appropriate stability testing are water‐based drug products packaged in semipermeable containers. In addition to the routine stability tests outlined previously, these products also require tests to demonstrate that water loss under conditions of low relative humidity do not occur.

## 4 PHARMACOKINETICS, PHARMACODYNAMICS, AND PHARMACOGENETICS

Currently, the mechanistic evidence that is always systematically evaluated in the drug approval process consists of studies of pharmacokinetics (PK) and pharmacodynamics (PD). Below, we briefly describe the roles of these, together with the closely related field of pharmacogenetics. Pharmacokinetics is the study of how a drug enters, distributes within, and clears the body. Pharmacodynamics is the study of how varying concentrations of the drug in the body produce therapeutic and adverse effects. Pharmacogenetics is the study of the genetic influences on drug pharmacokinetics and pharmacodynamics. Together, these sciences provide insights into the complex‐systems mechanism(s) that influence the concentration of the drug in the body and the relationship between the concentration of the drug and the drug's effects. Knowledge of a drug's pharmacokinetics, pharmacodynamics, and pharmacogenetics is rarely complete, but rather accumulates throughout drug development and subsequent clinical use. Pharmacokinetic and pharmacodynamic considerations are vital for making a number of key decisions during drug development. Specifically: What range of doses should be used at first in human studies? Which dose or doses should be selected for testing in phase III pivotal studies? What are the potential adverse effects of the drug and adverse reactions to it? For these reasons, PK and PD are among the types of mechanistic evidence required for a proper evaluation and interpretation of trial evidence. At these points, evidence from experimental animal models is used to estimate the likely response in humans. How reliable this extrapolation is depends on the relevant similarities between humans and the animal models used, comparable to evaluating external validity of trials (see Section 8. External validity).

All three sciences, pharmacokinetics, pharmacodynamics, and pharmacogenetics, are important for deciding which patients should receive the drug and what doses should be used in different patients. All three sciences have developed rapidly over the past two decades. The clinical applicability of pharmacogenetics in particular is recent and is likely to play an increasingly significant role in clinical drug development, regulation, and clinical use. Concrete examples of the roles these sciences play in providing evidence for and from mechanisms for drug evaluation are provided below.

## 5 DEVISING DOSAGE REGIMENS

**Figure 1:**
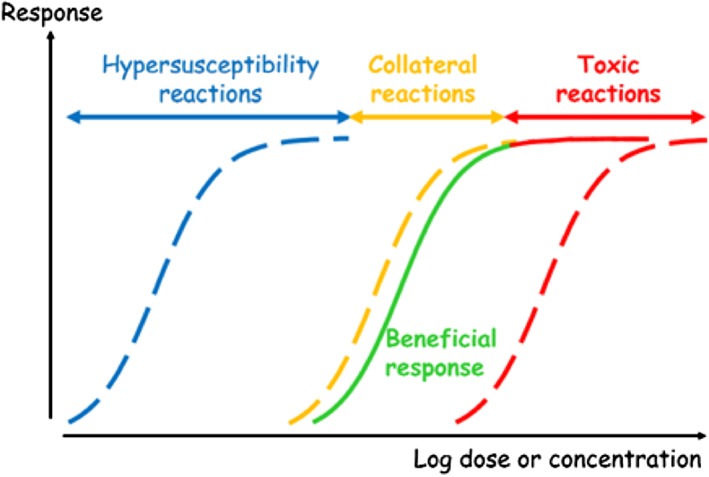
Classification of adverse drug reactions according to their dose relations (see the text for explanation)

The development of appropriate dosage recommendations provides an excellent example of the “learn‐confirm” cycles that occur throughout clinical drug development and clinical use of the drug. Much work early in clinical drug development is focused on determining the drug's dose‐response relationship (see Figure 1). In early‐phase trials, this will be informed by the drug's pharmacology (pharmacodynamics), pharmacokinetic studies in healthy volunteers, and dose‐ranging studies. Dose‐ranging studies seek to identify the smallest dose that produces a measurable effect on an outcome of interest (the “minimum effective dose”) and the “maximum tolerated dose” (doses above which adverse effects occurred that required withdrawal of the drug in the majority of patients). This preliminary understanding of the drug's dose‐response relationship is enhanced as further insight about the drug's pharmacokinetics and pharmacodynamics is gained in studies on an increasingly wide variety of patients.

An area of considerable importance is being able to explain and predict how patient demographics, physiology, and/or genetics influence how the drug is absorbed, distributed within the body, and then eliminated. Increasingly sophisticated approaches to pharmacokinetic modelling are being used throughout drug development to identify appropriate dosage regimens for testing in pivotal trials and subsequent clinical use. Physiologically based pharmacokinetic (PBPK) modelling is an example of these approaches. PBPK explicitly starts with a structural model based on the complex systems mechanisms involved in absorbing, distributing, and eliminating drugs.^10^

Understanding the mechanisms that explain a drug's pharmacokinetics remains important for determining appropriate doses for individual patients following drug approval. For example, the approved dosage recommendations for the anticoagulant enoxaparin in the treatment of deep vein thrombosis are typically stated relative to body weight. This is because elimination of enoxaparin is influenced by renal function and metabolism, which tend to vary in predictable ways with body weight. The challenge, however, is that lean body weight is a better predictor of the clearance of enoxaparin than total body weight. The distinction is unimportant in the leaner patients that are often enrolled in clinical trials, but critical in the broader range of patients treated in routine care.^11^, ^12^ Dosing an obese patient using total body weight rather than lean body weight puts the patient at risk of toxicity. Understanding the mechanisms of enoxaparin's elimination informs appropriate dosing; a drug that is distributed throughout the body differently or eliminated differently will require a different approach.

## 6 EFFICACY

For approval, a drug must be shown to be efficacious in patients with the targeted disease or condition. Phase III (pivotal) trials are meant to demonstrate this sufficiently well to merit licensing. Demonstrating efficacy requires showing that the treatment is correlated with improvement in the condition, and that any observed difference between the treatment and control groups is attributable to the treatment. The latter requires sufficient evidence for ruling out explanations of the correlation in terms of chance, bias, or confounding, so that the only remaining explanation is that there is a mechanism linking the intervention and the outcome that shows how the former is at least partly responsible for the latter. An ideally conducted trial would provide this evidence directly: if a sufficiently large correlation were observed in a perfectly randomized, perfectly representative, sufficiently large trial, that would provide very strong evidence that the correlation is causal, ie, that there is some mechanism of action that gives rise to the correlation. In practice, however, studies tend to be imperfect in various respects and so less conclusive. In such cases, it can be useful to consider the evidence in favour of the hypothesised mechanism of action. A well‐established mechanism of action can support the efficacy claim, while a hypothesised mechanism that has little evidence or contrary evidence (ie, lack of biological plausibility) can undermine the efficacy claim.

At present, mechanistic considerations tend to be treated rather unsystematically at drug approval meetings. Often “the evidence” is taken to consist of reports of phase III trials, which are selected and analysed in detail in advance of the approval meeting, and subjected to further scrutiny at the meeting. On the other hand, discussion of mechanisms occurs principally at the meeting itself, mediated through the opinions of the experts and without its role or relevance being clear to all participants. The fact that evidence for mechanisms is part of the evidence base and can be crucial to evaluating efficacy is not widely recognized. However, such evidence can be analysed as systematically as evidence from phase III trials.^13^

One obvious example of the crucial role for evidence for mechanisms in judgements of efficacy occurs when determining biosimilarity. A biosimilar is a biological medicine that is very similar to another biological medicine that has already been approved for use. Often the burden of proof in phase III trials is much lower for biosimilar drugs than for other drugs. Instead, those assessing biosimilarity rely more on evidence of similarity of mechanism of action, particularly evidence of similarity of structure and function.^14^ For example, http://www.ema.europa.eu/docs/en_GB/document_library/Summary_of_opinion_-_Initial_authorisation/human/003916/WC500216075.pdf, a treatment for osteoporosis with active ingredient teriparatide, was http://www.ema.europa.eu/ema/index.jsp?curl=pages/medicines/human/medicines/003916/human_med_002060.jsp&mid=WC0b01ac058001d124 by the European Medicines Agency (EMA) without a major new study, on the grounds of biosimilarity with http://www.ema.europa.eu/ema/index.jsp?curl=pages/medicines/human/medicines/000425/human_med_000798.jsp&mid=WC0b01ac058001d124, a different formulation of teriparatide.^15^, ^16^

While biosimilarity refers to similarity of complex biological molecules, bioequivalence refers to *in vivo* biological similarity of different formulations of the same compound, typically small molecules. Again, the burden of proof in the terms of clinical trials is lowered, subject to appropriate mechanistic evidence: principally, evidence of pharmaceutical and pharmacokinetic properties of the two formulations.^17^, ^18^

Standards of proof are different to that for biosimilarity, because small molecules are accurately reproducible, and two formulations of the same medication will contain exactly the same active molecule, even though inactive excipients may differ. On the other hand, complex molecules are sensitive to small differences in product engineering, which may affect the pharmacokinetics and pharmacodynamics of the compound. Biological medicines are typically more complex than 100% synthetic compounds, and so extra scrutiny is required in establishing biosimilarity. Biosimilarity can depend on the whole manufacturing process starting from the choice and engineering of the *in vitro* system to purification, not just the drug.

Evidence for mechanisms also informs judgements of efficacy when evaluating the design of clinical studies and the appropriateness of the inferences drawn from their results. Determining whether a study design is of high quality and is based on sound science requires evidence for mechanisms, notably when assessing the diagnostic categories used in a study, whether the length of the trial was appropriate to demonstrate efficacy, and whether all plausible confounders were controlled for.^3^ When clinical studies are found to be defective, evidence for mechanisms may be used as grounds to motivate requests for new studies.

As an example of the use of evidence for mechanisms to assess the inferences drawn from clinical studies, consider the case of quetiapine. A well‐accepted standard for assessing the efficacy of an antidepressant is to conduct a randomized trial and compare the effects of the antidepressant against placebo on the sum‐score of a depression rating scale. Commonly used rating scales include the Hamilton Depression Rating Scale and the Montgomery‐Åsberg Depression Rating Scale. Each of these scales consists of items assessing common symptoms of depression, including feelings of sadness and effects on sleep and appetite. There is increasing recognition of the limitations of focussing on sum‐scores in assessing depression and response to treatment.^19^, ^20^ The approval of quetiapine for use in major depressive disorder highlights the importance of evaluating mechanisms in addition to accepted methodological standards when assessing drug efficacy.

Quetiapine is an antipsychotic drug that was originally developed for the management of schizophrenia but has also been licensed for use in major depressive episodes in bipolar disorder (as “add‐on” or “adjunctive treatment” in the USA and UK and as monotherapy in Australia). The application for approval was supported by several trials of quetiapine in patients with major depressive disorder. These trials met the methodological requirements for assessing antidepressant efficacy. However, mechanistic considerations raise a number of questions regarding the validity of the accepted standard for assessing antidepressant efficacy. Most relevant is the effect of quetiapine on sleep. Two trials report the effects of quetiapine on individual Montgomery‐Åsberg Depression Rating Scale items.^21^, ^22^ The largest effect of quetiapine by a substantial margin is its effect on sleep. Sedation and somnolence were also the most commonly reported adverse reactions, occurring in 25% to 40% of participants taking quetiapine (compared with 6% of those taking placebo). This raises the possibility that quetiapine's effect on sleep leads to an over‐estimate of its efficacy as an antidepressant. This is further supported by the quicker than expected efficacy of quetiapine observed in the trials, as early as the first week.^21^, ^22^ If a sedative effect influences the apparent efficacy of quetiapine, that would explain the quick response.

The development of dalcetrapib provides a striking case of the interplay between evidence for mechanisms and evidence from clinical trials when assessing efficacy. Dalcetrapib causes an increase in high density lipoprotein (HDL) cholesterol concentrations. The properties of HDL cholesterol and low density lipoprotein (LDL) cholesterol are complex, but relatively well understood.^23^ In broad terms, LDL cholesterol *promotes* atherosclerosis through the formation of fatty plaques in the arterial wall, and HDL cholesterol *prevents* atherosclerosis by facilitating the removal of cholesterol from the arterial wall. The epidemiological evidence is consistent with this understanding: people with higher concentrations of LDL cholesterol tend to have higher rates of cardiovascular disease and people with higher concentrations of HDL cholesterol tend to have lower rates. Furthermore, there is very strong evidence from clinical trials that drugs that lower LDL cholesterol, such as statins, reduce the risk of cardiovascular disease. The same effect, however, has not been demonstrated for drugs that raise HDL cholesterol without affecting LDL.

After showing promising results in early clinical development, dalcetrapib failed to demonstrate efficacy in a large phase III study.^24^ This trial randomized 15 871 patients at high risk of cardiovascular disease to dalcetrapib or placebo. Dalcetrapib increased HDL cholesterol concentrations, but the study was terminated once it was clear that participants who took dalcetrapib were no less likely to experience a cardiovascular event. The lack of efficacy of dalcetrapib in reducing cardiovascular disease brought into question the mechanistic evidence regarding the anti‐atherosclerotic properties of HDL cholesterol. These findings implied that the mechanisms linking HDL cholesterol and cardiovascular disease were more complex than had been thought.^3^ However, there is an alternative mechanistic explanation for the observed lack of efficacy of dalcetrapib. Tardif et al conducted a genome‐wide association study using data from clinical trials, including the failed clinical trial.^25^ They identified a single nucleotide polymorphism associated with a response to dalcetrapib. Dalcetrapib benefited participants with one version of the gene and harmed participants with another version. The first group of participants had a 39% reduction in cardiovascular events while taking dalcetrapib; the second group had a 27% increase in cardiovascular events. Thus, the disjunction between the original mechanistic hypothesis and the subsequent trial evidence led to a modified mechanistic hypothesis that a subgroup of individuals might benefit. This new mechanistic hypothesis is being tested in a phase III clinical study in participants with the polymorphism, which seeks to show that dalcetrapib is associated with beneficial outcomes in this group.^26^

## 7 HARMS (ADVERSE EFFECTS AND REACTIONS)

All efficacious drugs have targets by which they produce benefit. Targets are usually tissue proteins, such as membrane‐bound or intracellular receptors, ion transporters or channels, and enzymes. A few drugs are used as replacements for deficient or absent endogenous substances, such as hormones (eg, levothyroxine), minerals (eg, iron), vitamins (eg, vitamin B_12_), and enzymes (eg, pancreatic enzymes). In some cases, the therapeutic target is not known but must exist; for example, the therapeutic target for lithium is not known, although the enzyme inositol‐1‐phosphatase, which it inhibits, is a strong candidate.

Adverse effects of drugs are also produced by actions on targets. In some cases, the target is the same as that by which the beneficial effect is produced; such effects are called “on‐target effects”. However, most adverse effects are produced by actions on targets other than those that produce benefit; these are called “off‐target effects”. The principles are illustrated in relation to the dose‐related classification of adverse drug reactions (Figure 1).

In Figure 1, each curve is a theoretical dose‐response (concentration‐effect) curve. Adverse drug reactions follow three patterns in relation to the dose‐responsiveness of the beneficial effect (in green)^27^^:^

- hypersusceptibility reactions (blue), in which the reactions occur at doses or concentrations lower than those associated with benefit;
- collateral reactions (orange), in which the reactions occur at doses or concentrations in the same range as those associated with benefit;
- toxic reactions (red), in which the reactions occur at doses or concentrations higher than those associated with benefit, either through the same mechanism (solid line) or some other mechanism (dotted line).

The solid lines show on‐target effects, the dotted lines off‐target effects.

Apart from adverse reactions that occur through exaggeration of the target effect (ie, some toxic reactions; red solid line in Figure 1), all adverse reactions are off‐target. For example, bleeding due to the anticoagulant warfarin is an on‐target reaction, due to excess anticoagulation. Such reactions can be dealt with by reducing the dose, and dosage regimen calculations (see Section 5) take this into account. To understand all other adverse reactions, it is necessary to understand the mechanisms by which they occur, which will not be the mechanisms whereby the benefits occur.

Animal studies can be useful. For example, some drugs can cause cardiac arrhythmias in association with prolongation of the electrocardiographic QT interval, which represents the time between the start of depolarization of the ventricles and full repolarization (ie, contraction and relaxation). The mechanism by which this prolongation occurs is through inhibition of the cardiac potassium channels known as human ether‐a‐go‐go‐related gene channels. Knowing this, it is now routine practice for all drugs to be screened for inhibitory effects on human ether‐a‐go‐go‐related gene channels using, for example, sheep cardiac Purkinje fibres in vitro. If the outcome is positive, the drug is not further developed.

Pharmacogenetic mechanisms can contribute to the risks of adverse reactions. For example, there are interindividual variations in the activities of enzymes of the CYP family, depending on polymorphisms in the relevant genes. Knowing in advance the pharmacokinetic mechanisms whereby a drug is metabolized allows predictions about adverse drug interactions and risks of adverse reactions in particular populations. Similarly, pharmacodynamic polymorphisms can help predict the risks of adverse reactions to some medicines.

Dimethyl fumarate (Skilarence), a treatment for psoriasis, was considered by the Commission on Human Medicines (CHM) of the UK Medicines and Healthcare products Regulatory Agency (MHRA) on 24 March 2016. It concluded that the mechanisms of action (anti‐inflammatory and immunomodulating effects) were well established, but that further mechanistic studies were needed to investigate the risk of harms, particularly because carcinogenicity had been observed in animals. In addition, mechanistic evidence suggest that dimethyl fumarate alters lymphocyte function and (by extrapolation from treatments for multiple sclerosis) may increase susceptibility to progressive multifocal leukoencephalopathy, a fatal viral inflammatory disease of the brain caused by the JC virus (named after a patient, John Cunningham), which if present in the body can be reactivated when immune function is impaired, for example by medication. Skilarence was eventually approved by the Committee for Medicinal Products for Human Use (CHMP) of the EMA in April 2017.^28^

## 8 EXTERNAL VALIDITY

For a drug to be approved, the regulator must assess the external validity of the trial evidence submitted in support of the benefit to harm balance of the candidate drug: can the results obtained in a trial population be applied to the population for which the drug is to be licensed? In the ideal case, the trial and target populations are the same or similar in most respects, so that no differences between the populations can be expected. This is very rarely the case, but if in doubt, the regulators may require more clinical trials with more representative samples to be conducted before approval.

The problem of external validity is compounded when the target population is, for example, small children, pregnant women, or patients with comorbidities. These groups are not typically included in clinical trials for ethical reasons, or, in the case of comorbidities, because of potential confounding by their inclusion. In such cases, the same features that prevent the use of clinical trials in specific patient populations consequently make those patients highly dissimilar to the average trial subjects and therefore vitiate external validity. One must then use means other than clinical trials to assess whether those dissimilar features of the target population will make the patients react differently to the drug, possibly undermining assessment of the benefit to harm balance.

Mechanistic evidence features crucially in evaluating external validity of trials. Differences in the mechanisms underlying the effects of a drug, or in features that could interfere with the mechanisms, can undermine the application of the trial results to the target population.^29^ Thus, for the application of the trial results to the target population to be warranted, one must provide evidence that the relevant mechanisms and factors capable of interfering with the mechanisms are similar in the trial and the target populations. This involves more than just considering the biological plausibility of the drug's efficacy; one must provide some positive evidence for the relevant similarities. These similarities can be established in various ways, either through consulting evidence that directly demonstrates similarity of features of mechanisms, or by observing that similar outcomes persist across populations expected to vary in the features of the mechanism, or by inferring the mechanism's similarity based on evidence from closely related populations. Parkkinen et al (2018) provide a more detailed description of how the evaluation of mechanistic similarities may proceed.^13^

Abaloparatide is a treatment for osteoporosis in post‐menopausal women who have a high risk of fractures. It was considered by the CHM on 24 March 2016. The argument centred on extrapolation: a clinical trial showed that abaloparatide reduced the risk of vertebral fractures;^30^ however, although the mechanism of action would apparently also apply to non‐vertebral fractures, it was considered that there was insignificant evidence for extrapolation. This led to a request for further research. Abaloparatide was eventually approved by the FDA in the USA in April 2017, but remained under https://www.sps.nhs.uk/medicines/abaloparatide/ by the CHMP as of July 2017.^31^ The CHM raised concerns that abaloparatide, which is intended for use in older women, had been tested only in healthy women, and raised concerns about the most frail patients. Grounds for concern included the fact that half of all patients in a trial developed anti‐abaloparatide antibodies and that abaloparatide injection led to a marked increase in heart rate.

Sofosbuvir/velpatasvir is a combination therapy for hepatitis C. The CHM considered this treatment on 24 March 2016 but raised a safety concern on the grounds of extrapolation: velpatasvir has been found to cause serious teratogenicity across three species (mouse, rat, and rabbit), and this robustness across species was thought to provide significant evidence of a possible teratogenic effect in humans. Robustness of effect is important evidence of similarity of mechanism.

## 9 COST EFFECTIVENESS

If the benefit to harm balance of a medication is acceptable, and the manufacturer receives a licence to market it, there remains the question of whether a health care system can afford to use it to treat members of its population, given that health care budgets are limited. One way of deciding this is to calculate the cost‐effectiveness of the medication, ie, whether the effect it offers gives good value for money. The usual method for doing this is to calculate the overall cost of using the medication and dividing it by a measure of the quality of life that is gained by using it. The quality of life is assessed by a measurement called the quality adjusted life year or QALY. A QALY of 1 implies perfect health and a QALY of 0 implies no health at all (ie, death). QALYs are typically measured using instruments that elicit patients' answers to questions about their health. For example, one such instrument, the EQ5D, asks how problematic the individual finds mobility, self‐care, usual activities, pain/discomfort, and anxiety/depression. The difference between the QALYs before and after treatment, the QALY gain, is divided into the cost, giving an incremental cost‐effectiveness ratio (ICER). In the UK, if an intervention has an ICER of £20 000 to £30 000 per QALY gained, it is considered to be cost‐effective and can be recommended for funding by the health care system.

Mechanisms are often not discussed by committees charged with determining the cost‐effectiveness of therapeutic interventions, but understanding mechanisms can influence decisions in various ways.

In constructing pharmacoeconomic models that relate clinical outcomes to costs, it may be helpful to include mechanistic considerations. For example, in a multiple comparison of different types of antihypertensive drugs a decision will have to be made about whether to compare drugs with different mechanisms of action (eg, beta‐blockers, diuretics, calcium channel blockers) and whether, within a pharmacological class, to include compounds with variable actions (eg, in the class of beta‐blockers whether to compare full antagonists with partial agonists).^32^ The cost‐effectiveness of rituximab has been studied using a mechanism‐based pharmacoeconomic model that included population pharmacokinetics and pharmacodynamics, linking serum rituximab concentrations to progression‐free survival, simulating the effectiveness of rituximab in various clinical contexts.^33^ These mechanisms served as inputs to economic models of follicular lymphoma, based on NICE appraisals.

If an intervention is claimed to be efficacious but the proposed mechanism of action is not biologically plausible, or is biologically implausible, or if there is no well attested mechanism, the claim of efficacy may be vitiated and the size of the QALY gain put in doubt.

In some cases, conflicting analyses can be informed by an appeal to mechanisms. For example, in an indirect comparison of two medications that both increased platelet counts in children with idiopathic thrombocytopenia, an analysis by the manufacturer of one of the medications suggested that there was no significant difference between the two compounds, while an independent analysis suggested otherwise.^34^ The fact that the two treatments had different actions on the thrombopoietin receptor mediating platelet synthesis suggested that there was likely to be a difference, supporting the results of independent analysis. Although the data were too poor for a firm conclusion to be made about the size of the difference, this mechanistic argument, when taken with other considerations, helped the appraisal committee to reach a decision.

## 10 ADHERENCE

The reasons people seek treatments, the reasons they adhere to the treatments offered, and the interaction between help seeking and subsequent adherence to treatment have been extensively investigated over many decades.^35^, ^36^, ^37^, ^38^, ^39^ Factors affecting adherence include individual patient characteristics and the doctor‐patient relationship as an interacting complex system. Sometimes, adherence and non‐adherence are conceptualized as opposite sides of the same coin—the opposite of doing something being not doing something. Sometimes, they are seen as quite different behaviours—doing something positive (adherence) or doing something else positive (non‐adherence). There is also a series of sub‐specialities in the social sciences which attend to the behaviours and the behaviour changes involved. Much of this is empirically based.

The human actions involved take place in a social milieu that varies widely in terms of infrastructures (such as hospitals, primary care settings, and pharmacies), the competencies of staff and patients, and the meanings that people attribute to what is going on.^40^ In order to fully describe the mechanisms of effectiveness in real‐life settings, these features have to be an intrinsic part of the explanation, along with the biological mechanisms.

The method traditionally used to deal with poor adherence is intention to treat analysis. This is the statistical device of including all people who entered a trial in the final statistical analysis, even if they dropped out, stopped talking the medication, or took it variably. This gives a proxy for how well the drug will work in the total population on average. It is outcome focussed. This is of course better than simply assuming that what works under controlled laboratory or clinical settings will work in the field. But it skates past the behavioural mechanisms that are involved and remains locked into looking at associations, correlations, and averages in total populations, not at mechanisms.

It follows from this that in the drug approval process there is a set of mechanisms that involve human behaviour. Just as the sciences of pharmacokinetics and pharmacodynamics constitute an intrinsic part of the process of approval, so too should the sciences of psychology and sociology, along with epidemiological and statistical methods and intelligence about industrial manufacture, quality control, and packaging. Formally, with the exception of the behavioural sciences, these all constitute parts of the decision‐making process for drug approval. What needs to be guarded against is a default to common sense assumptions or various forms of heuristic thinking about behavioural matters because that inevitably leads to bias and mistakes.^41^, ^42^

Despite many efforts to understand the mechanisms underlying poor adherence to medications and to improve it, adherence continues to pose a major problem in the interpretation of data in pivotal trials during drug development. Variable adherence can have major consequences in clinical trials, resulting in confusion in their interpretation and reduced accuracy of estimates of benefit and harms. This can adversely affect drug development, especially of treatments that have a narrow therapeutic window. The consequences can include failure to confirm efficacy, underestimation of efficacy or of the risks of harms, emergence of drug‐resistant organisms in trials of anti‐infective agents, and impaired development of breakthrough drugs and drugs for rare diseases.^43^ Better understanding of the mechanisms of poor adherence from psychological and sociological studies could help mitigate these problems, and the use of a mechanistic taxonomy in studies of adherence should be routine.^44^ Sponsors of trials should be required to declare how adherence was measured and to what extent it was achieved.

## 11 EXTENDING PRODUCT LICENCES

Extending a product licence (marketing authorization) to new indications and/or new populations requires regulatory approval. The following examples illustrate the difficulties of assessing licence extension and the role that evidence for mechanisms and inferences based on presumed mechanisms play in assessing the desirability of extension.

Extending the indication of a treatment already on the market typically requires new clinical trial evidence of the benefit to harm balance of the treatment for the new indication. The importance of this is illustrated in cases in which treatments have been adopted for new populations or indications that have subsequently been shown to be ineffective or insufficiently safe. The use of antidepressants in adolescents follows this outline. Before 2005, antidepressants were used extensively in adolescents with depression. This practice rested, at least in part, on the assumption that the benefit to harm balance in adolescents would be similar to that seen in clinical trials in adults with depression. While this remains controversial, systematic reviews and evidence made available through legal cases have brought into question the efficacy and safety of several antidepressants in adolescents, as well as the reporting of several sponsor‐initiated studies.^45^, ^46^ By 2005, regulatory bodies in the USA, UK, and Europe had issued warnings regarding the risk of increased suicidal behaviours associated with antidepressants.

The extent to which antidepressants were prescribed for adolescents in the belief that the benefit to harm balance was the same as in adults represents an example of failed extrapolation. This could have been addressed by waiting for compelling evidence from clinical trials before use. However, this response is not entirely satisfactory, given the challenges of treating depression and the continuing uncertainty regarding harms, specifically the risk of suicidal ideation. Questions remain about appropriate measures of suicide risk, when suicide and attempted suicide are attributable to treatment, and there is evidence of poor data practices, publication bias, and, as has been suggested in some cases, data manipulation in relation to suicide risk.^46^, ^47^ There is also uncertainty regarding whether or not the risk of suicidal ideation associated with antidepressant use varies with age.^47^ While evidence for mechanisms plays an important role in debates regarding appropriate measures, there is very little mechanistic evidence in this case that helps to adjudicate between key areas of the debate. For instance, there is very little mechanistic evidence to help adjudicate whether the risk of suicidal ideation is expected to differ between adults and adolescents using antidepressants.

In other cases, mechanistic evidence may be available. The recent extension of the licence for ivacaftor in cystic fibrosis provides a striking example. In May 2017, the FDA expanded the licence for ivacaftor to several new genetic variants of cystic fibrosis. Cystic fibrosis results from a defective transmembrane conductance regulator (CFTR) gene, which encodes a protein that facilitates the transport of salt and water in and out of cells. Specific mutations that either block the CFTR gene from being transcribed or affect the protein product in particular ways will cause loss of functionality, generating the clinical manifestations of cystic fibrosis. The nature of the symptoms varies, depending on which CFTR allele is affected. Different drugs that work to compensate the defect therefore vary in their effectiveness against different cystic fibrosis genotypes. Many of the mutations are so rare that conducting clinical trials to test the effectiveness of a drug against specific genotypes would be impractical, if not impossible. Instead, in its decision, the FDA relied extensively on *in vitro* evidence that provides insight into the disease mechanism. Because the functional defect—loss of regulation of ion and water transport—is known, and the mechanisms responsible for it are fairly well characterized, in vitro assays demonstrating that cells regain function in the presence of a drug are expected to provide a good biomarker of clinical success. Laboratory evidence of this effect in different CFTR mutant cells, together with trial evidence for previously approved indications, allowed the FDA to conclude that the drug will work in several cystic fibrosis genotypes not tested in clinical trials.^48^ Such use of mechanistic evidence requires more than considering the biological plausibility of a treatment. Rather, one must explicitly evaluate the evidence that speaks to the operation of the mechanism, and the evidence must be of good quality.

## 12 DISCUSSION AND RECOMMENDATIONS

Evidence‐based medicine seeks to make evidence explicit and to develop explicit methods for evaluating it. In practice, present‐day EBM focuses almost exclusively on clinical studies—it treats mechanistic evidence that arises from other sources as irrelevant or peripheral. But mechanistic evidence is neither of those things: we have argued that evidence for mechanisms, ie, evidence that mechanisms exist and how they operate, is central to drug approval, because it informs the drug approval process in a wide variety of ways. We believe that the drug approval process would benefit from explicitly including mechanistic evidence as part of the assessment of manufacturers' applications for licences and in postmarketing surveillance, so that it can be appropriately scrutinized and, if need be, challenged. The EBM+ programme explores ways of making evidence of mechanisms explicit and suggests ways of using such evidence to inform causal evaluation (see, for example, ref 10). Therefore, this paper can be viewed as motivating a broadening of the evidence base of EBM.

While Parkkinen et al^13^ consider the epistemological role of evidence for mechanisms in some detail, they do not make specific procedural recommendations for committee‐based structures for drug approval. Our recommendations would be as follows. At present, on behalf of a committee for drug approval (such as that of the UK Medicines and Healthcare products Regulatory Agency), dedicated professionals produce a detailed analysis of certain factors that inform drug approval decisions, such as the quality and statistical features of the clinical trials and the quality of the manufactured drug. This analysis is then presented to the committee for discussion. We suggest that, in addition, the hypothesised mechanisms of drug action and best clinical use should be assessed in advance of the drug approval meeting and this analysis should be presented to the committee members. This analysis should highlight key features of the mechanisms that are not established and should include an assessment of the evidence for these contentious features. The person responsible for this task would need general clinical pharmacological training, in order to be able to quickly identify what is already well established and does not require scrutiny of evidence. This person would also need some familiarity with typical social mechanisms related to clinical use. If this mechanistic summary were provided to the panel well in advance of the approval meeting, there would be an opportunity to get feedback from the clinicians on the committee, to ensure that all the relevant questions were addressed and the relevant evidence made available, and that no contentious features of the relevant mechanisms were overlooked.
